# Anaesthesia Considerations on Paediatric Neurosurgery

**DOI:** 10.4274/TJAR.2024.241698

**Published:** 2025-03-21

**Authors:** Rudin Domi, Filadelfo Coniglione, Asead Abdyli, Gentian Huti, Krenar Lilaj, Federico Bilotta

**Affiliations:** 1 Medical University of Tirana, Department of Anaesthesiology and Intensive Care, Tirana, Albania; 2 Tor Vergata University of Rome, Rome, Italy; 3 “Sapienza” University of Rome, Department of Anaesthesiology, Critical Care, and Pain Medicine, Rome, Italy

**Keywords:** Bleeding, neurosurgery, neurotoxicity, paediatric anaesthesia, positioning

## Abstract

Paediatric neurosurgery has seen significant increases and improvements because of advancements in technology and monitoring techniques. This type of surgery presents unique challenges to the anaesthesiology team because of the general characteristics of paediatric patients and the complexity of the procedures. Managing paediatric patients undergoing complex neurosurgery requires profound knowledge of age-related normal physiology and the principles of common paediatric neuroanaesthesia. This review focuses on updated information about various critical topics in paediatric neurophysiology, bleeding management, acute pain treatment, intraoperative neuromonitoring, the specifics of the sitting position, and the general principles of paediatric neuroanaesthesia.

**Main Points**

• Paediatric neurosurgical anaesthesia requires dedicated staff with comprehensive and profound knowledge.

• Specific “hot” points include paediatric neurophysiology, tumor-specific characteristics, intraoperative bleeding, sitting position, postoperative pain treatment, neuromonitoring, and extubation-related differences in neurosurgery.

• Advances in monitoring and endoscopic surgery have improved patient prognosis.

## Introduction

Paediatric neuroanaesthesia presents a unique challenge to paediatric and adult neuroanaesthesiologist. Children undergoing neurosurgical procedures have different physiological and morphological features, making this type of surgery and patient population very special.^1^ Children present with specific challenges in physiology, pharmacology, anaesthesia care, intensive care unit (ICU) monitoring and treatments, and neurological follow-up. Nowadays, paediatric neurosurgery is becoming more common in practice, so every anaesthesiologist must be aware of its important specifics and the skills required.^2^ Neuroanaesthesia principles in children are the same as in adults and include neuromonitoring, decreased perioperative intracranial pressure, brain tissue oxygenation and perfusion, adequate haemodynamics, and early evaluation after the procedure.^3^ There are several key challenges in neurosurgical anaesthesia care that anaesthesiologists must address. Childhood tumors are often localized in the posterior fossa, thereby making the sitting position and its consequences a concern. Anatomical and physiological parameters vary with the age of the child. Perioperative neurological evaluation presents age-related difficulties owing to poor communication. Important aspects such as airway management, vascular access, anaesthesia induction, anaesthesia maintenance, blood loss, and recovery from anaesthesia differ significantly between neurosurgical procedures in adults and children. Table 1 summarizes the most important anaesthesia care concepts for paediatric neurosurgery. This review provides updated knowledge on paediatric neurosurgical anaesthesia, with a focus on new developments.

### Neurological and Haemodynamic Features of Paediatric Patients

In paediatric neurosurgery, the clinical scenario requires careful attention to both paediatric-specific physiology and the intricacies of neurosurgical care, demanding a tailored approach from the anaesthesiologist. Children, particularly infants and younger age groups, present unique challenges due to developmental differences in cerebral hemodynamics. Baseline cerebral blood flow and autoregulation parameters are generally lower in paediatric patients than in adults, although these values progressively increase and align with adult norms as children age. Autoregulation, typically within a range of 20-60 mmHg in paediatric patients, can be easily disrupted, necessitating careful blood pressure management to maintain stable cerebral perfusion.

A significant factor to consider is the elevated cerebral metabolic rate of oxygen (CMRO₂) in children, which makes them more vulnerable to adverse effects from hypoxia, hypotension, and hypoglycemia.^4^ These risks stem from children’s high metabolic demands and limited physiological reserve compared with adults. Consequently, any compromise in oxygen supply can rapidly lead to cerebral ischemia and neuronal injury. Therefore, anaesthesiologists must vigilantly monitor oxygenation and circulation to prevent hypotension and hypoxemia. This involves careful titration of anaesthetic agents, fluid management, and frequent assessment of hemodynamic status.

Additionally, the anaesthesiologist must be familiar with age-specific normal ranges for vital signs because children’s heart rates and blood pressure significantly vary across developmental stages. Precise control of these parameters is essential to maintain optimal cerebral perfusion and minimize the risk of intraoperative complications.^4^ Understanding these age-related variations is critical in adapting anaesthesia plans to support neurological outcomes in paediatric neurosurgery.

### Anaesthetic Technical Challenges in the Paediatric Patients

Preoperative evaluation is a critical element in paediatric neuroanaesthesia. Depending on the child’s age, the anaesthesiologist may encounter challenges in obtaining information directly from the patient, making the input from relatives essential. Another significant aspect of the procedure is assessing the patient’s level of consciousness and potential increased intracranial pressure. Preoperative neurologic evaluation is crucial for documenting any existing deficits.^5^ If the patient has a congenital disease, other associated congenital conditions may be present, necessitating a cardiac evaluation. Additionally, it is important to carefully assess the volume status and dehydration to optimize preoperative vascular bed filling.^6^

**Figure 1:**
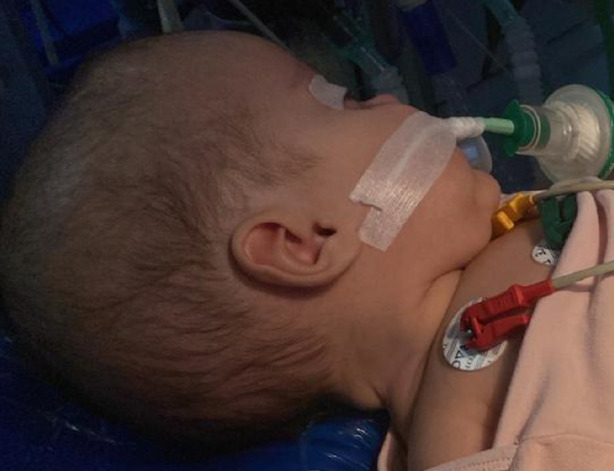
Large head in a hydrocephalus baby (original photo)

**Figure 2:**
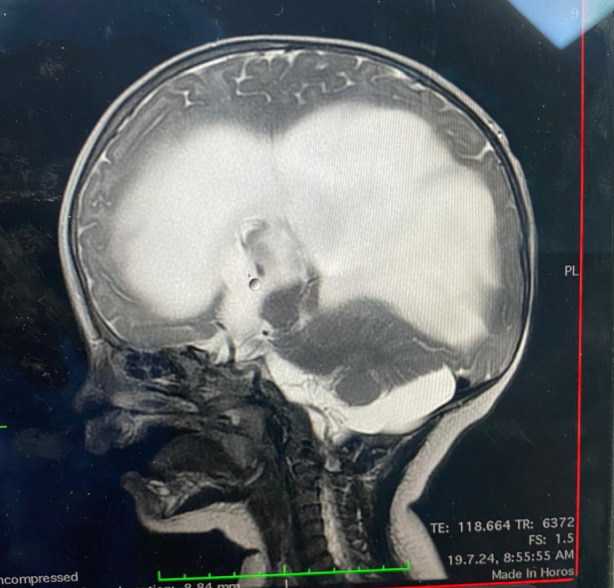
Characteristic imaging images of a patient with hydrocephalus (original photo)

**Figure 3:**
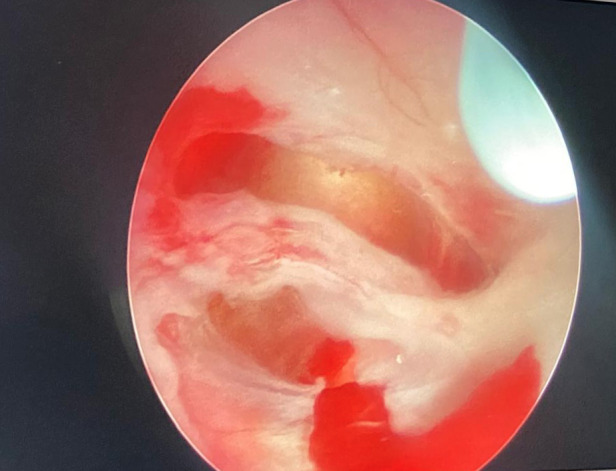
Intraoperative endoscopic treatment views (original photo)

**Figure 4:**
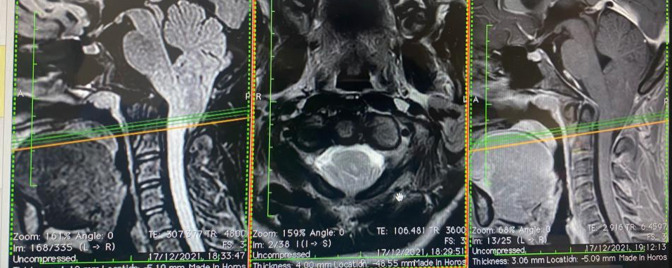
Chiari malformation (original photo)

Airway and vascular access management can be challenging. If the patient is agitated, inhalation sedation is suitable for inserting a peripheral venous cannula; however, it is not recommended for patients with reduced consciousness who require rapid induction sequence to prevent aspiration. Central lines are generally inserted when a peripheral cannula is not feasible or significant volume and blood transfusion are anticipated. The femoral route for the central line may be suitable and must be removed asap to minimize thrombosis. The echo-guided insertion approach is a common practice and increases procedure success and safety.^7^ Airway management and intubation are critical steps in caring for these patients. Babies with hydrocephalus (Figures 1, 2, 3) often have increased head circumference, thereby complicating ventilation and intubation. In cases of Chiari malformation (Figure 4), any head movement during ventilation and intubation can lead to brainstem damage.^8^ Patients with craniosynostosis may experience temporomandibular joint ankylosis, which is associated with reduced mouth opening and intubation difficulties.^8, 9^ Further increases in intracranial pressure, hypoxemia, severe hypotension, and gastric aspiration must be avoided as much as possible.

Anaesthesia maintenance can be done using inhalator anaesthetic agents, total intravenous anaesthesia, or an inhalator-intravenous combination approach. Sevoflurane appears to be safer for paediatric patients than for adults because it does not cause significant cerebral vasodilation. Sevoflurane is used not only for sedation to obtain vascular access but also for anaesthesia maintenance. Total intravenous anaesthesia is preferred when vascular access is already established in the ward and the patient is not agitated, allowing for easier manipulation. No data show a clear advantage of one technique over the other.^10^

Emergency anaesthesia and child extubation requires special attention and is often associated with complications mainly respiratory.^11^ Paediatric neurosurgery is considered an intermediate-risk procedure for extubation because of an increased risk of reintubation due to impaired airway and respiratory control.^11, 12^ Two techniques have been reported: awake extubation and sleep extubation.^13^ Extubation after paediatric neurosurgery differs significantly from extubation after other paediatric surgeries. Several predictors of delayed extubation in children undergoing neurosurgical procedures have been proposed. Various authors have identified factors such as preoperative mental status, surgery duration exceeding six hours, extensive resection, cranial nerve damage, brain edema, hypothermia, and significant blood loss as predictors of delayed extubation.^14^ Sangtongjaraskul et al.^15^ conducted a study involving 539 paediatric neurosurgical patients and found a 10% incidence of delayed extubation. The primary causes of this delay were blood loss exceeding 40% of the total blood volume, preoperative oxygenation status, and intracranial surgery.^15^ Thus, extubation after paediatric neurosurgery may present challenges due to not only general paediatric complications but also decreased consciousness, new postoperative deficits, cranial nerve damage, and neurosurgical postoperative complications, such as cerebral edema, pneumocephalus, and intracranial bleeding.

### Anaesthetic Physiological Challenges in Paediatric Patients

**Figure 5:**
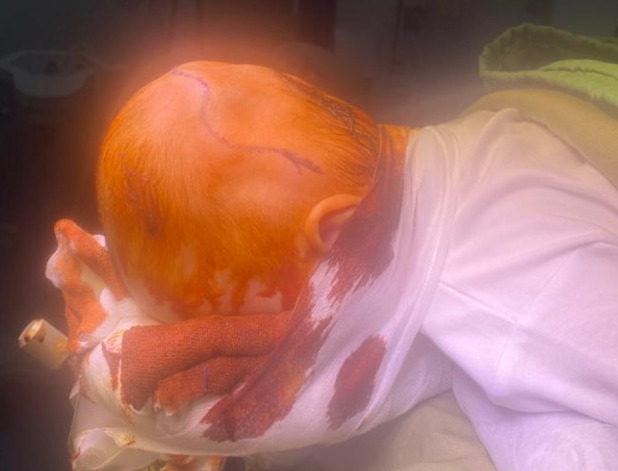
Patient in the prone position undergoing craniosynostosis surgical correction (original photo)

**Figure 6:**
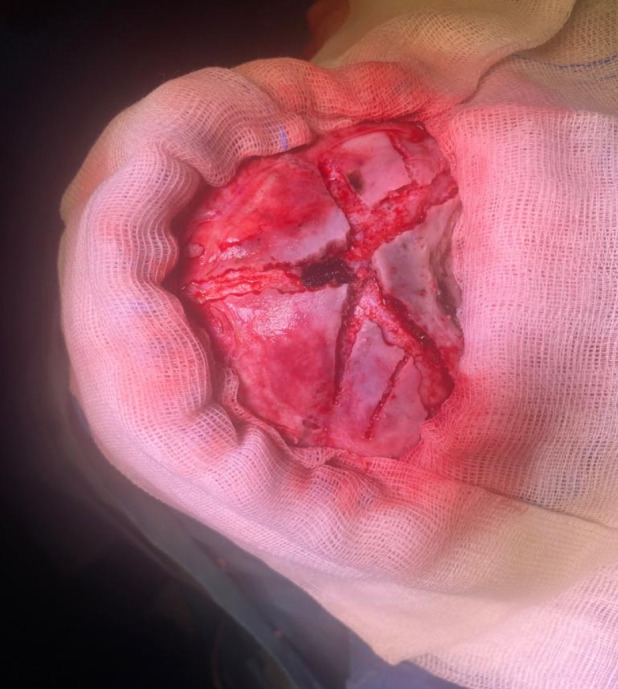
Surgical correction of craniosynostosis (original photo)

Intraoperative bleeding during paediatric neurosurgery often occurs during specific procedures, such as craniosynostosis (Figures 5, 6), but can also occur in large tumor resections. The principles of management are the same as those in adults and include hemodynamic and hypoperfusion management, as well as the prevention of coagulopathy. In children, blood loss may be insidious and can seriously compromise tissue perfusion. It is essential to evaluate blood loss according to age, weight, brain condition, and preoperative hemoglobin level.^16^ Blood transfusions are more frequently associated with complications (allergy, hemolysis) than in adults.^17^ Recognizing risk factors is crucial for developing a detailed management approach. Recently, several predictor factors for poor prognosis have been reported, including low weight, large tumors, prolonged surgical duration, and perioperative anaemia.^18^ The volume of bleeding is generally correlated with hemodynamic disturbances, reduced brain perfusion, reduced tissue oxygenation, a large volume of fluid administration, and complications from blood transfusions.^19^ A hemoglobin level >8 g dL^-1^ may ensure normal cerebral tissue oxygenation.^20^ King et al.^21^ reported data from 6,583 patients who underwent craniosynostosis surgery and found no side effects of tranexamic acid, such as seizures or thrombosis. Several studies have reported that tranexamic acid is effective in reducing intraoperative bleeding and the need for transfusions.^22, 23^ de Faria et al.^24^ found no benefits of tranexamic acid in brain surgery, but noted its effectiveness in brain and spine trauma. Interesting results have been published by Goobie et al.^25^ They compared the effects of low and high concentrations of tranexamic acid. They concluded that a loading dose of 10 mg kg^-1^, followed by a maintenance dose of 5-10 mg kg^-1^ h^-1^, could reduce the need for transfusions without adverse effects.

Pain treatment following neurosurgery is a cornerstone in postoperative period. It is generally accepted that opioids for acute pain treatment after neurosurgery may be effective, with close monitoring of side effects. Non-steroidal anti-inflammatory drugs are often used as adjuvants because of the risk of bleeding, even in the absence of evidence. However, Xing et al.^26^ published data on 320 paediatric neurosurgical patients and found that opioids such as tramadol, fentanyl, and morphine may be safe. Multimodal analgesia has gained popularity in recent years. A meta-analysis by Kulikov et al.^27^ recently reported the efficacy of this analgesic technique and the use of regional analgesia after neurosurgery. The systematic review (PROSPECT) was published, including 53 randomized controlled trials.^28^ The authors found that multimodal analgesia, combining non-steroidal anti-inflammatory drugs, dexmedetomidine, paracetamol, and scalp blocks, was effective in treating acute postoperative pain. They concluded that opioids should be considered if non-opioid treatments fail. Regional analgesia is now performed in many centers as part of multimodal analgesia.^29^ This can reduce systemic drug administration and side effects. It is important to consider patient characteristics and the type of neurosurgical procedure when selecting the appropriate analgesia regimen.

Intraoperative neuromonitoring is crucial in various neurosurgical procedures. The most common neuromonitoring techniques are motor evoked potentials and somatosensory evoked potentials. These techniques enhance the quality of the procedure, ensure patient safety, and reduce the risk of further brain damage.^30^ Several intraoperative factors, including hypotension, hypoglycemia, antiepileptic drugs, inhalants, and muscle relaxant use, can affect neuromonitoring results. Inhalators (sevoflurane, isoflurane, nitrous oxide) can trigger epileptic episodes, especially if the minimum alveolar concentration is >0.5, and can modulate motor-evoked potential results.^30, 31^ Total intravenous anaesthesia appears to be safe. If the use of muscle relaxants is necessary, the anaesthesiologist may choose a short-acting agent and monitor neuromuscular blockade using TOF. Thus, in small infants, the anaesthesiologist can start with inhalational sedation, secure vascular access, and continue with total intravenous anaesthesia after intubating the patient’s trachea without muscle relaxants.

**Figure 7:**
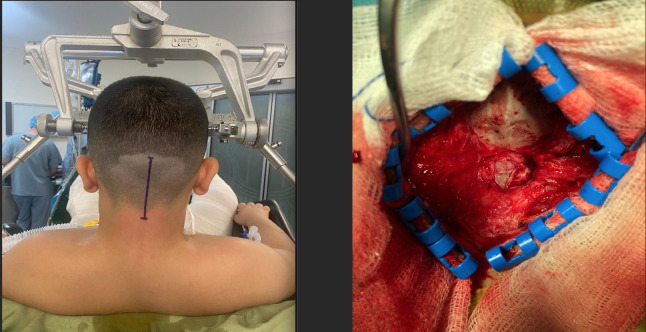
Patient in the sitting position for posterior fossa surgery and scull exposure (original photo)

**Figure 8:**
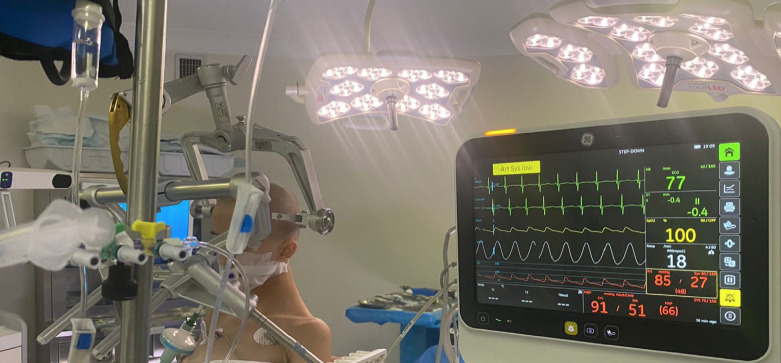
Arterial line transducer positioned in the external auditory meatus for accurate invasive blood pressure monitoring (original photo)

The sitting position is often used in children because brain tumors are predominantly located in the posterior fossa (Figure 7). Surgeons may prefer the sitting position based on their personal preferences and institutional protocols. This position offers surgical advantages, including better exposure through direct access and improved drainage of blood and cerebrospinal fluid. However, this approach introduces several physiological changes that are of particular concern to anaesthesiologists. Complications associated with the sitting position include hypotension, severe jugular vein obstruction, brain edema, facial and pharyngeal edema, and venous air embolism. Hypotension can result from gravitational pooling of blood in the abdomen, reduced venous return, preoperative hypovolemia due to mannitol, vomiting, or fasting, use of positive end-expiratory pressure to prevent venous air embolism, and the vasodilatory effects of anaesthetics.^32^ To manage hypotension, the anaesthesiologist must optimize vascular bed filling by administering fluids, using intermittent pneumatic compression stockings, administering vasopressors, and correcting the patient’s position. To ensure adequate cerebral perfusion, the arterial invasive monitoring transducer should be positioned at the level of the external auditory meatus (Figure 8). Venous air embolism is a significant complication associated with the sitting position.^33^ This typically occurs when the surgical site is approximately 10 cm above the right atrium, allowing venous air to enter the right atrium. This can cause air embolism, right heart failure, impaired cardiac output, hypotension, hypoperfusion, and potentially death if not promptly and aggressively treated, especially if a large amount of air (5 mL kg^-1^) enters.

Sudden decreases in ETCO_2_, hemodynamic disturbances (such as hypotension and arrhythmias), and hypoxemia strongly suggest venous air embolism. The diagnosis is confirmed by echocardiography (either transthoracic or transesophageal). Treatment includes reversing the patient’s position, aspirating air from a central catheter (if one has been previously inserted), administering fluids, and using vasopressors or inotrope to support hemodynamics.^33^

Several studies have examined the incidence of venous air embolism in children. Bithal et al.^34^ found that the incidence and severity of venous air embolism in children are comparable to those in adults when they are in the sitting position. The authors concluded that the sitting position is safe for children undergoing posterior fossa surgery. Harrison et al.^35^ published data for 16 years of experience, reporting a 9.3% incidence of venous air embolism with no perioperative consequences. In a retrospective analysis, Dilmen et al.^36^ included 601 adults and 91 children who underwent surgery in the sitting position. They reported an incidence of venous air embolism of 20.4% in adults and 26.3% in children, with no related complications. Thus, they found the sitting position to be safe for both adults and children.^36^ Teping et al.^37^ studied the semi-sitting position in paediatric neurosurgery and reported their data for 10 years of experience. They enrolled 42 patients who underwent posterior fossa surgery and found an 11.9% incidence of venous air embolism, but without hemodynamic instability. The authors concluded that the semi-sitting position is safe if performed by a dedicated and experienced anaesthesiology staff. Therefore, every paediatric anaesthesiologist or neuroanaesthesiologist must have profound knowledge and experience regarding the physiological consequences of the sitting position to ensure safe and successful posterior fossa surgery.

## Conclusion

Paediatric neurosurgical anaesthesia requires a dedicated and experienced staff. A multidisciplinary team, including paediatricians, neurologists, neurosurgeons, anaesthesiologists, and nurses, can ensure patient safety and improve treatment outcomes. In addition to new developments in paediatric research, a profound understanding of the physiological and anatomical features of paediatric patients is crucial.

## Figures and Tables

**Table 1. Specific anaesthesia concepts for common paediatric neurosurgical procedures:** 

**Brain tumors**	• Prone/sitting/supine position • Hormonal/non-hormonal secreting • Brainstem tumor (bradycardia, cardiac arrest) • Cranial nerve damage (especially mixed nerves) • Increased intracranial pressure • Maintain adequate MAP and CPP • Avoid hypotension and hypercapnia • Delayed extubation • Reinforced endotracheal tube • Endotracheal tube position (head movement during positioning) • Diabetes insipidus in craniopharyngiomas (hypernatremia, polyuria) • Sodium balance during the third and lateral ventricles
**Hydrocephaly**	• Open vs. endoscopic • Local vs. general anaesthesia (depend on age, metal status, and procedure) • If external drainage: local anaesthesia may be performed • If available, local anaesthesia can minimize hypotension, hypoxia, and delayed extubation. • Hypothermia prevention (warm fluids and blankets) • Arrhythmias resulting from ventricular distention • Bradycardia (from increased intracranial pressure, fast and large cerebrospinal fluid amount evacuation) • Antibiotic prophylaxis
**Craniosynostosis**	• Other abnormalities (cardiac, Crouzon, Apert, Pfeiffer, metabolic) • Careful preoperative cardiac evaluation • Increased intracranial pressure (if hydrocephaly associated) • Difficulty in airway management (large head, temporomandibular joint stiffness) • Increased incidence of bleeding, hypothermia, and infections
**Chiari malformations**	• Brainstem compression • Difficulty/careful airway management • Severe bradycardia/cardiac arrest • Sitting position (hypotension, venous air embolism)
**Epilepsy surgery**	• Chronic antiepileptic therapy • Neurodevelopment problems • Increased liver metabolism (larger anaesthetic dose) • Inhalators may be epileptogenic • Unexplained tachycardia may include seizures • Intraoperative seizures (propofol, lorazepam, local iced water irrigation)
**Encephalocele**	• Microcephaly and external herniation • Careful positioning (accidentally rupture) • Other congenital malformations • Difficult airways • Antibiotic prophylaxis
**Meningomyelocele**	• Often associated with Chiari type 2 • Careful airway manipulation (brainstem stimulation) • Antibiotic prophylaxis • Bleeding • Hypothermia

MAP, mean arterial pressure; CPP, cerebral perfusion pressure.
